# Electrochemotherapy Is Effective in the Treatment of Bone Metastases

**DOI:** 10.3390/curroncol29030139

**Published:** 2022-03-04

**Authors:** Laura Campanacci, Luca Cevolani, Francesca De Terlizzi, Laura Saenz, Nikolin Alì, Giuseppe Bianchi, Davide Maria Donati

**Affiliations:** 1 Unit of 3rd Orthopaedic and Traumatologic Clinic Prevalently Oncologic, IRCCS Istituto Ortopedico Rizzoli, Via Pupilli 1, 40136 Bologna, Italy; laura.campanacci@ior.it (L.C.); nikolin.ali@ior.it (N.A.); giuseppe.bianchi@ior.it (G.B.); davidemaria.donati@ior.it (D.M.D.); 2 IGEA Clinical Biophysics, Via Parmenide 10/A, 41012 Carpi, Italy; f.deterlizzi@igeamedical.com; 3 Orthopedic Department, National Children’s Hospital, Calle 20 Av 0, San José 10103, Costa Rica; lsaenzm@ccss.sa.cr

**Keywords:** electrochemotherapy, bone metastases, quality of life, minimal-invasive treatment

## Abstract

Bone metastases induce pain, risk of fracture, and neural compression, and reduced mobility and quality of life. Electrochemotherapy (ECT) is a minimally invasive local treatment based on a high-voltage electric pulse combined with an anticancer drug. Preclinical and clinical studies have supported the use of ECT in patients with metastatic bone disease, demonstrating that it does not damage the mineral structure of the bone and its regenerative capacity, and that is feasible and efficient for the treatment of bone metastases. Since 2009, 88 patients with bone metastasis have received ECT at the Rizzoli Institute. 2014 saw the start of a registry of patients with bone metastases treated with ECT, whose data are recorded in a shared database. We share the Rizzoli Institute experience of 38 patients treated with ECT for a bone metastasis, excluding patients not included in the registry (before 2014) and those treated with bone fixation. Mean follow-up was 2 months (1–52). Response to treatment using RECIST criteria was 29% objective responses, 59% stable disease, and 16% progressive disease. Using PERCIST, the response was 36% OR, 14% SD, and 50% PD with no significant differences between the two criteria. A significant decrease in pain and better quality of life was observed at FU.

## 1. Introduction

According to a Global Cancer Observatory (GLOBOCAN) 2020 publication, cancer is one of the leading death causes worldwide and the most significant disease burden globally [1]. In 2019, Mattizu et al. published an article reviewing cancer epidemiology; overall, a little more than 18 million new cancer cases were diagnosed in 2018 [2].

Although this scenario could be perceived as discouraging, since 1970, with the implementation of chemotherapy as adjuvant treatment along with radiotherapy and surgical resection, significant steps towards disease control have occurred. However, because of survival times prolonging and cancer rates increasing due to demographic changes, accumulative cancer cases are rising [3]. This compound number of cancer patients has forced medical care providers to widen treatment options to improve quality of life.

Many complications that patients with cancer might experience and compromise their life quality arise from bone metastasis [4]. Symptoms such as pain, spinal cord compression resulting in neurological deterioration, metabolic imbalances secondary to hypercalcemia, and prolonged movement reduction due to pathological fracture or risk of fracture must be handled. Bone is the third most frequent target tissue for metastases. Lung, prostate, breast, kidney, and thyroid cancers are responsible for almost 80% of these skeleton lesions [5,6]. The treatment of metastatic patients is multidisciplinary. Systemic therapies are essential, but local treatments must sometimes be added to solve or prevent orthopedic complications.

Standard local treatments include radiation therapy, surgery, embolization, focused ultrasound treatment, or cryotherapy [7,8,9,10]. Electrochemotherapy (ECT) is a minimally invasive local treatment based on the combination of high-voltage electric pulses and anticancer drugs. Because of its proven efficacy for treating cutaneous and subcutaneous tumors, its application has also been extended for internal tumors. Preclinical and clinical studies supported the use of ECT in patients with metastatic bone disease, demonstrating that it does not damage the mineral structure of the bone and its regenerative capacity. Moreover, clinical trials demonstrated the feasibility and efficacy of electrochemotherapy for the treatment of bone metastases [11,12].

Since 2009, 88 patients with bone metastasis have received ECT at the Rizzoli Institute. In 2014, a registry of patients with bone metastases treated with ECT was started. Since then, all patient data are recorded in a shared database (http://reinbone.wng.it accessed on 15 February 2022) protected by security passwords.

This paper aims to share the Rizzoli Institute experience of 38 patients that received ECT to treat bone metastases whose data were registered in the database. Eighteen patients undergoing fixation after ECT were excluded. This decision was under the premise that somehow bone reaction to intramedullary nailing procedure could overlap with ECT response when assessing bone healing.

## 2. Materials and Methods

From 2014 to 2021, 56 patients with bone metastasis received ECT at the Rizzoli Orthopedic Institute. This study was approved by local Ethical Committee (Comitato Etico di Area Vasta Emilia Centro della Regione Emilia-Romagna (CE-AVEC) Protocollo Generale 0028598, 25 August 2014). Inclusion criteria for ECT treatment were as follows: age >18 years, and histologically proven involvement of appendicular or axial skeleton by metastatic carcinoma or melanoma. Exclusion criteria were as follows: coagulation disorders, severe pulmonary edema or fibrosis, pregnancy or lactation, known allergy to Bleomycin or cumulative dose exceeding 400,000 IU, and chronic renal dysfunction. For this paper, 18 patients undergoing fixation after ECT were excluded; also, one patient died prior to achieving the intended length of follow-up.

Prior to data gathering, ethics committee and data protection authority approval was requested. Then, for the remaining group of 38 patients, data concerning age, diagnosis, previous treatments, metastases localization, ECT parameters, and clinical outcome were recorded. Imaging evaluation to assess tumoral morphology included conventional X-ray and MRI, CT with contrast enhancement, or FDG-PET scans.

The electroporation system used was the Cliniporator^®^ VITAE (IGEA S.p.A., Carpi, Italy). Hardware included trocar-type electrodes of diameter 1.8 mm. Length selection was according to localization, morphology, and dept of the metastasis. Available electrodes had a total length to choose between 12 to 20 cm composed of two parts, one insulated for soft tissue protection and the other considered the “active part” non-insulated, which might be 30 or 40 mm. Electrode diameter, length, and positioning were selected according to careful pre-operative planning to accomplish configuration requirements for effective electroporation.

Using diagnostic radiological images, the lesion to be treated was modeled using specific PULSAR planning software (C3M, Centre for Computational Continuum Mechanics, Ljubljana, Slovenia). Treatment planning was done considering the size and location of individual tumors, with respect to major blood vessels and preferred electrode insertion geometry, so that the number and the geometric distribution of the electrodes, their distances, pairs of electrodes for the pulse the delivery and the pulses for each pair of electrodes were calculated accurately verified. This procedure has made it possible to determine the optimal positioning of the electrodes to ensure complete and homogeneous electroporation of the tumor mass.

ECT procedure was carried out following ESOPE guidelines [13]. Eight pulses of 1000 V/cm between electrode pairs delivered a homogenous electric field over the lesion. Several numerical models of electric field generated by needle electrodes have been investigated and are available in the literature [14], showing that the electric field distribution is optimal when the active part of the electrodes are placed inside the site of treatment, i.e., the bone lesion. In this case, the electric field generated inside this material (which is homogeneous from the electric characteristics point of view) can be easily predicted. If the electrodes are correctly inserted into the tumor lesion, the surrounding bone and muscles will not affect the electric field distribution inside the lesion. Furthermore, device feedback analysis of the electric current flowed during the treatment can eventually adjust the intensity of the electric pulse and assure the highest coverage of the lesion with a sufficient intensity of electric field.

Electrochemotherapy requires covering the entire tumor with a pulsed electric field, the distribution of which depends on the biology of the treated tissues.

The Cliniporator calculates the most appropriate electric field to achieve electroporation in all of the treated tissues; after the delivery of the current, the Cliniporator communicates if electric field between the pairs of electrodes was effective in inducing the electroporation of the tissue crossed by the current; if not, the machine recalculates the parameters of the current, and the electrical supply with the new parameters is then repeated. In the case of particularly complex or highly uneven lesions, pre-treatment planning can be a good solution to optimize the procedure.

Bleomycin was the selected chemotherapeutic with a dosage of 15 mg/m^2^ of body surface (Bleomycin Nippon Kayaku, Sanofi Aventis, Milan, Italy).

After patients underwent peripheral blocking or general anesthesia, electrodes were positioned with imaging assistance, using either fluoroscopy or CT scan. Then, intravenous Bleomycin bolus was administrated, and 8 min were waited to allow drug distribution. Next, electroporation was immediately performed to meet the 30-min time-frame limit after Bleomycin injection to be efficacious.

Evaluation of clinical and radiological response was performed at two timeframes, the first one within 60 to 90 days after ECT and the second one, among surviving patients, at 6 months. For clinical evaluation, quantifying tests were submitted to analyze data. The Visual Numeric Scale was used to evaluate pain [15], while the EQ-5D-3L questionnaire and ECOG Scale of Performance Status was applied to assess overall performance [16]. Additionally, to assistance in rating tumoral response, PERCIST and RECIST criteria was used when possible [17,18].

Continuous variables were described by median value and range, mean and standard deviation. Categorical variables were described by absolute number and percentage. The relationship between each criterion of response was assessed using a Chi-square test or, where appropriate, Fischer exact test. Logistic univariate analysis was performed to identify clinical or instrumental variables that could influence the objective response rate among those registered in the database; relative risk (RR) and *p* value have been reported. Data were statistically analyzed using the Mann–Whitney U test used for nonparametric analyses. Statistical significance was defined as *p*\0.05. All analyses were performed with IBM SPSS version 21.0 (IBM Co., Armonk, NY, USA).

## 3. Results

A total of 38 patients met our inclusion criteria. The median age of the 38 patients was 59 years (range 41–91 years) and the median time since diagnosis of the primary tumor was 49 months (range 0–226 months). Table 1 summarizes the descriptive statistic of the cohort of patients.

Thirty-seven patients underwent a single ECT session, while one underwent two ECT sessions, for a total amount of 39 ECT sessions. According to tumor size and geometry, the number of needles used for each session varied from 3 to 11. For correct electrode positioning, CT scan guidance was used in 16 cases and fluoroscopy in 22. Detailed information regarding ECT sessions and treated lesions are reported in Table 2. In two cases, the lesion was located near the joint plane (acetabulum), and in these, the treatment with ECT did not cause injury to the joint.

The most treated sites were the long bones of the limbs and the pelvis. Treatment with variable-geometry electrodes requires keeping the active tips of the electrodes parallel to each other, and this limits the use of ECT in the vertebral sites, for which it is necessary to insert the electrodes trans-peduncularly. However, in 2019, Cornelis et al. [19] showed that it is possible to perform ECT in vertebral sites, especially the lumbar. Due to the proximity of the pleura to the ribs, the risk of causing pleuro-pulmonary complications with the insertion of the electrodes has led to us not performing this treatment in the costal sites.

The total procedure lasted from 20 to 80 min (median 40 min) depending on the size of the target lesion. The median follow-up time was 2.2 months (range 1–52 months) with a mean value of 6.6 ± 4.9 months. One out of 38 patients died before the first follow-up due to the rapid worsening of the systemic conditions, and was excluded. Therefore, follow-up was available for 37 patients, and 15 of them (39%) achieved a follow-up longer than three months with an average of 12.3 ± 14.5 months.

One patient presented early skin necrosis with bone exposure after the treatment, requiring amputation. The patient was a 91-year-old suffering from squamous cell carcinoma of the forearm. He underwent mass excision and local flap coverage. He was referred due to a further recurrence with tumor invasion of the proximal 2/3 of the ulna. Given the extent of the lesion, arm amputation was proposed. The patient refused the amputation, so ECT was performed with custom geometry electrodes to treat the skin tumor and its extension into the bone. However, treatment-induced necrosis caused bone exposure, eventually requiring amputation.

The response to treatment is summarized in Table 3.

Overall, the response to treatment by RECIST criteria comprised nine objective responses (25%), three complete responses (9%), and six partial (16%). Twenty-two patients had stable disease (59%) and six a progressive disease (16%).

According to PERCIST criteria, 13 objective responses (36%) were recorded, of which five were complete (14%) and eight partial (22%). Five patients had stable disease (14%) and 19 a progressive disease (50%). No significant differences were observed between the two criteria (*p* = 0.44). The univariate logistic model revealed that gender, age, histology, number of metastases, and fractures did not influence the outcome. Only ECOG 0–1 is a predictor of objective response in this cohort of patients (*p* < 0.0001). Analyzing the radiological result based on the type of lesion (lytic, sclerotic or mixed), of the 29 lytic lesions, three had a CR, five a PR, 16 SD and four PD (one case was not evaluable due to death within one month). The sclerotic lesion remained radiologically stable; of the eight mixed lesions, one showed a partial radiological response, five remained stable and two progressed. Twenty-three of the 29 patients with osteolytic lesion were taking anti-resorption drugs. The radiological outcome of these patients was not considered to be affected by this therapy, because it has been ongoing for many months prior to ECT treatment. Regarding pain intensity before ECT, four patients (11%) reported no pain, three patients (8%) had mild pain, 18 (47%) moderate, and 13 (34%) had severe pain, with a median value of 6 ± 2.7. All four patients in whom ECT treatment was performed in the absence of pain had a lytic lesion at the femoral neck, a site where, in the event of growth of the lesion, the risk of pathological fracture increases with subsequent indication for surgical hip replacement. Treatment in these cases was therefore performed to prevent major complications with minimally invasive treatment. Additionally, the analysis of pain management in these patients revealed that before ECT, four patients (11%) did not require any painkillers because they had no pain. Six patients (16%) sometimes used analgesics, another six (16%) continuously used non-opioids drugs, 18 (47%) required opioids, and three (8%) had uncontrolled pain (one unknown).

After ECT, from the 37 evaluable patients, 25 showed pain reduction (68%). Ten patients did not experience any change in their pain status (including the four patients with no pain before ECT), and two experienced worsening pain.

To summarize pain response, 17 out of 37 patients (45%) reduced painkillers after the ECT with a significant improvement of pharmacologic pain management (*p* = 0.0049).

A significant decrease in pain intensity was observed at the mean Visual Numeric Scale (VNS) after ECT at early (2 ± 2.7) and late (2 ± 2.6) follow-up visits concerning pre-ECT values (*p* < 0.0001 and *p* = 0.0046, respectively).

**Figure 1:**
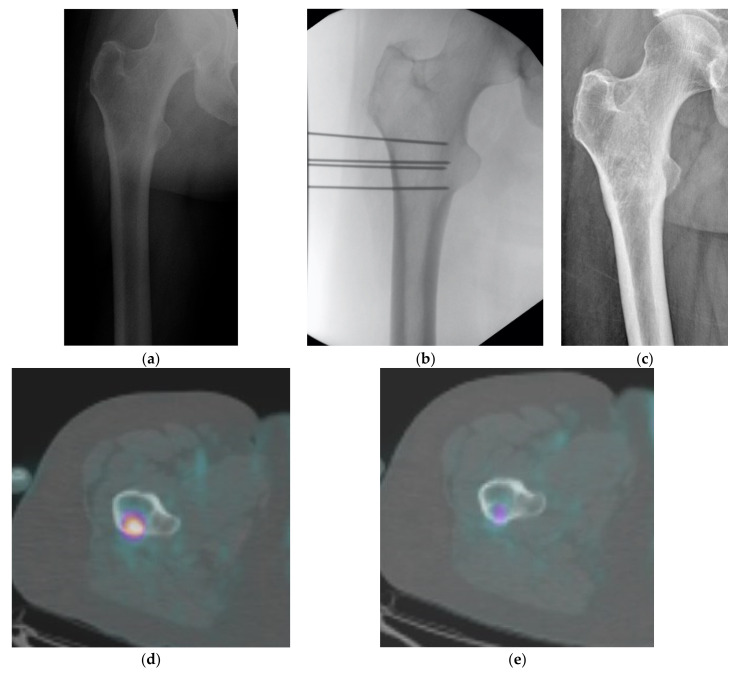
(**a**–**e**) Woman 47 years old. Osteolytic lesions in proximal femur in bone metastasis from breast carcinoma treated with ECT. (**a**) Pre-op X-ray, (**b**) intraoperative fluoroscopic check, (**c**) 18 months of follow-up: pain decreased from VNS 9 to 0/10 and the lesion partially ossified. PET scan performed before (**d**) and 6 months after treatment (**e**) shows a partial metabolic response of the lesion.

Correlating pain with X-ray imaging, among the 25 patients with pain improvement, seven experienced substantial bone recovery, two complete, and five partial. The other 17 patients remained without changes, and one presented disease progression. Amid the four patients with no pain (pre- and post-ECT), bone quality improved in two, and the other two remain without changes. Additionally, among the eight patients with stable or worsened pain, three had no X-ray changes, and five displayed disease progression (Figure 1).

Twelve patients performed local radiation therapy one year before ECT at least (1 ± 0.8 years). One patient resulted in CR, three PR, seven SD and one PD. Eight out of 12 patients improve their performance status.

Finally, there are no significant trends in EQ-5D items, probably due to the low number of cases, except for the pain item (*p* = 0.0450). However, although not significantly, it is clear that the quality-of-life condition improved at follow-up compared with the pre-ECT status. The univariate logistic model revealed that ECOG values 0–1 were significantly associated with a higher objective response rate (*p* < 0.0001).

## 4. Discussion

Metastatic bone disease is a significant health-care issue, affecting 4.9 million individuals in the United States [20]. Prostate, breast, lung, kidney, and thyroid cancers account for approximately 80% of cases.

The main goals in metastatic bone patients are pain control and preventing local disease progression that may lead to a pathological fracture. Presently, except in the obviously moribund patient, it is still not possible to accurately assess remaining survival [9,21]. Natha et al. [22] analyzed the survival in patients treated for pathological fracture, and found that short and intermediate survivors were overestimated and long survivors were underestimated in terms of expected duration of survival.

Although the role of open surgical treatments with osteosynthesis for pathological fractures and/or resection of solitary metastases in long bones remains vital for independent ambulation and quality of life for these patients, minimally invasive techniques are safe with few complications and optimal results for cancer patients with bone metastases [23].

Presently, there is no single gold-standard treatment for bone metastasis, and deciding between all available options is still challenging. Commonly used therapies include systemic anticancer chemotherapies associated with local procedures (i.e., surgery, radiotherapy, percutaneous thermal ablation, cryoablation, bone cement, embolization, and focused ultrasound).

Surgery provides structural stability, but may be technically demanding and associated with prolonged recovery.

Radiotherapy (RT) is the most commonly used therapy to locally deal with bone metastases, with estimated rates of pain relief reported in the literature between 50% to 80%. Conventional external body RT is considered a non-invasive treatment but has complications inherent to tissue toxicity. Fibrosis, bone necrosis, or vascular insufficiency are somehow present to a certain extent in areas subjected to RT. This toxicity can potentially limit repetitive RT, often for 20% of patients under single-fraction and 8% of multi-fraction regimens. Additionally, it could compromise wound healing when future surgical procedures are required [24,25]. To mitigate some of these drawbacks and improve radiation delimitation, stereotactic RT has been introduced to treat bone metastasis. It is considered to enable higher-radiation doses while respecting neighboring structures [25]. 

To avoid RT side effects, other more recent technologies have been used. Regarding cryoablation, Callstrom et al. [26] reported a 75% response rate in 61 patients with painful bone metastases. Pusceddu et al. [27] described a 91% rate of improvement in the BPI scale at 12-week follow-up and 72% of patients free from symptomatology after microwave ablation.

Dupuy et al. [28] suggested the use of radiofrequency ablation for the treatment of painful bone metastases. In their patient series studied over a 3-month period, the authors observed a mean improvement of symptomatology of 26/100. The complication rate of these techniques is low, ranging between 0% (Pusceddu) and 5% (Dupuy). Bertrand et al. [29] reported PR 50% (8/16) and CR 37.5% (6/16) in patients treated with focused ultrasound. The use of transarterial embolization provide devascularization of the target area to guarantee safety and efficacy to the procedures carried out subsequently [30].

The common endpoint to all ablation techniques is the induction of the largest possible thermal necrosis of the target lesion to destroy periosteal nociceptors and reduce cancer size. There is no current evidence favoring the use of one ablation technique over another. In other hands, those techniques lead to a physical necrosis of the tumor cells, while ECT provides a chemical necrosis: in 2010, a study conducted by Fini et al. [31] demonstrated osteogenic activity and structural integrity of bone trabeculae after electroporation. Additionally, bone remodeling capability was preserved [31]. A few years later, in 2013, a preclinical in vivo and in vitro study showed that ECT could be safe, effective, and minimally invasive for treating bone metastases [11].

ECT does not induce bone necrosis, and therefore, in the case of fracture of the ECT-treated bone, healing is possible with usual fracture callus quality and healing time [12]. Between local treatments, ECT represents a good choice because it is minimally invasive and respects bone structure.

Pain is one of the most relevant quality-of-life indicators in patients who undergo palliative treatments. Pain relief was achieved in 68% of patients, and painkiller usage decreased in 45% of them. Our results show a significant pain reduction after ECT in more than half of patients at early follow-up. Moreover, we observed a reduction in pain even in the absence of an objective radiological response.

However, if we consider the radiological response (RECIST and PERCIST) by itself, it seems that the objective response to ECT is not very favorable. Nevertheless, among patients with pain improvement after ECT and those without pain before treatment (29 patients), the real radiological progression of disease was present in only one patient. Furthermore, objective changes in control imaging showed stable metastases behavior in 19 cases and improvement (complete or partial) in nine.

Complications related to ECT are usually few, predictable, and avoidable. In our patient cohort, complications associated with ECT were reported in only one patient, who developed tissue necrosis. The risk of skin necrosis and ulceration with further bone exposure in patients with poor soft tissue quality or previously irradiated areas was already described [12,32]. This complication can be diminished by inserting the active part of the electrodes beyond the epidermal layer avoiding skin damage, thus preventing bone exposure.

## 5. Conclusions

To conclude, ECT offers the possibility to use chemotherapy, a systemic treatment, in a localized fashion. Although it is not a complication-free treatment, drawbacks can be substantially decreased with meticulous pre-operative planning and careful case selection. In addition, conversely from other local treatments for bone metastases, ECT spares mineralized bone structure from irreversible damage, enabling bone healing. Finally, ECT is a valuable tool to alleviate pain and improve the quality of life for those patients facing the bone metastases burden.

## Figures and Tables

**Table 1:** Descriptive statistic of the population.

Descriptive Statistics on 38 Patients	N	%
SEX		
M	13	34%
F	25	66%
Primary tumor		
Breast	6	15%
Endometrial/uterus	6	15%
Lung	4	11%
Kidney	3	8%
Colon	4	11%
Vescica	3	8%
Liver	3	8%
Prostate	1	3%
Other *	8	21%
Presence of visceral metastases		
Yes	24	63%
No	14	37%
Pattern of metastatic disease		
Solitary bone mts	10	26%
Multiple bone mts	4	11%
Bone and visceral (non-lung)	8	22%
Bone and pulmonary	9	24%
Bone, visceral, pulmonary	7	17%
Pathologic fractures		
Yes	7	18%
No	31	82%
Type of lesions		
Lytic	29	76%
Sclerotic	1	3%
Mixed	8	21%
Previous treatments for metastases		
Chemotherapy	19	50%
Hormonal therapy	5	13%
Radiotherapy	12	32%
Other	6	16%
Performance status		
Fully active	3	8%
Restricted in physically strenuous activity	12	31%
Ambulatory capable but unable to work	16	42%
Capable of only limited self-care	6	16%
Completely disabled	1	3%

* SCC 1, GIST 1, nose 1, rectum 1, MPNST 1, sarcoma 1, adenoidocistic 1, liposarcoma 1.

**Table 2:** Detailed description of treated lesions and ECT parameters.

	N°	%	Median	Range	Mean ± St. Dev.
Lesion localization					
Lower limbs, of which					
Tibia	7	18%			
Femur	6	16%			
Tarsus	1	3%			
Upper limbs, of which					
Ulna	1	3%			
Scapula	3	8%			
Humerus	2	6%			
Trunk/pelvis, of which					
Iliac wing (+ pelvis)	10	25%			
Acetabulum	2	5%			
Sacrum	3	8%			
Pubic ramus	2	5%			
Costo-vertebral	1	3%			
Size					
Volume (cm^3^)			102	3.328 ± 138.2400	273.505 ± 34.9730
Axial direction (mm)			49	13–140	51 ± 27
Coronal direction (mm)			52	16–120	59 ± 29
Sagittal direction (mm)			50	16–120	58 ± 29
Duration of procedure (min)			42	18–80	46 ± 16
GUIDANCE					
Intensifier	22	58%			
CT	16	42%			
Number of electrodes used (39)					
3	2	5%			
4	8	21%			
5	5	13%			
6	19	50%			
7	1	3%			
8	1	3%			
11	2	5%			
Anesthesia					
Locoregional + deep sedation	31	82%			
General	4	10%			
Locoregional	2	5%			
Deep sedation	1	3%			
Completely covered lesion					
Yes	22	58%			
No	16	42%			
ECT sessions					
1	37	95%			
2	1	5%			

**Table 3:** Response to treatment according to RECIST and PERCIST criteria, and pain pre- and post-ECT (at early and at late follow-up).

	RECIST		PERCIST		Pain	Before ECT		Early FU		Late FU	
	N	%	N	%		N	%	N	%	N	%
**CR**	3	9%	5	14%	no	4	11%	8	27%	7	47%
**PR**	6	16%	8	22%	mild	3	8%	13	43%	5	33%
**SD**	22	59%	5	14%	moderate	18	47%	6	20%	1	7%
**PD**	6	16%	19	50%	severe	13	34%	3	10%	2	13%

CR = complete response; PR = partial response; SD = stable disease; PD = progressive disease; ECT = electrochemotherapy; FU = follow-up.

## Data Availability

http://reinbone.wng.it (accessed on 15 February 2022).
